# Burnout, depersonalization, and anxiety contribute to post‐traumatic stress in frontline health workers at COVID‐19 patient care, a follow‐up study

**DOI:** 10.1002/brb3.2007

**Published:** 2020-12-15

**Authors:** José Adán Miguel‐Puga, Davis Cooper‐Bribiesca, Francisco José Avelar‐Garnica, Luis Alejandro Sanchez‐Hurtado, Tania Colin‐Martínez, Eliseo Espinosa‐Poblano, Juan Carlos Anda‐Garay, Jorge Iván González‐Díaz, Oscar Bernardo Segura‐Santos, Luz Cristina Vital‐Arriaga, Kathrine Jáuregui‐Renaud

**Affiliations:** ^1^ Unidad de Investigación Médica en Otoneurología Instituto Mexicano del Seguro Social México; ^2^ Departamento de Psiquiatría del Hospital de Especialidades del Centro Medico Nacional siglo XXI Instituto Mexicano del Seguro Social Ciudad de México México; ^3^ Departamento de Imagenología del Hospital de Especialidades del Centro Medico Nacional siglo XXI Instituto Mexicano del Seguro Social Ciudad de México México; ^4^ Departamento de Terapia Intensiva del Hospital de Especialidades del Centro Medico Nacional siglo XXI Instituto Mexicano del Seguro Social Ciudad de México México; ^5^ Departamento de Admisión Continua del Hospital de Especialidades del Centro Medico Nacional siglo XXI Instituto Mexicano del Seguro Social Ciudad de México México; ^6^ Departamento de Inhaloterapia del Hospital de Especialidades del Centro Medico Nacional siglo XXI Instituto Mexicano del Seguro Social Ciudad de México México; ^7^ Departamento de Medicina Interna del Hospital de Especialidades del Centro Medico Nacional siglo XXI Instituto Mexicano del Seguro Social Ciudad de México México; ^8^ Laboratorio Clínico del Hospital de Especialidades del Centro Medico Nacional siglo XXI Instituto Mexicano del Seguro Social Ciudad de México México

**Keywords:** anxiety, burnout, depersonalization, post‐traumatic stress disorder

## Abstract

**Introduction:**

We designed a follow‐up study of frontline health workers at COVID‐19 patient care, within the same working conditions, to assess the influence of their general characteristics and pre‐existing anxiety/depression/dissociative symptoms and resilience on the development of symptoms of post‐traumatic stress disorder (PTSD), while monitoring their quality of sleep, depersonalization/derealization symptoms, acute stress, state anxiety, and burnout.

**Methods:**

In a Hospital reconfigured to address the surge of patients with COVID‐19, 204 frontline health workers accepted to participate. They completed validated questionnaires to assess mental health: before, during, and after the peak of inpatient admissions. After each evaluation, a psychiatrist reviewed the questionnaires, using the accepted criteria for each instrument. Correlations were assessed using multivariable and multivariate analyses, with a significance level of .05.

**Results:**

Compared to men, women reporting pre‐existing anxiety were more prone to acute stress; and younger age was related to both pre‐existent common psychological symptoms and less resilience. Overall the evaluations, sleep quality was bad on the majority of participants, with an increase during the epidemic crisis, while persistent burnout had influence on state anxiety, acute stress, and symptoms of depersonalization/derealization. PTSD symptoms were related to pre‐existent anxiety/depression and dissociative symptoms, as well as to acute stress and acute anxiety, and negatively related to resilience.

**Conclusions:**

Pre‐existent anxiety/depression, dissociative symptoms, and coexisting acute anxiety and acute stress contribute to PTSD symptoms. During an infectious outbreak, psychological screening could provide valuable information to prevent or mitigate against adverse psychological reactions by frontline healthcare workers caring for patients.

## Graphical abstract

During an infectious outbreak, pre‐existing burnout, dissociative experiences, and anxiety contribute to the development of post‐traumatic stress disorder in frontline health workers. To protect the mental health of vulnerable personnel and decrease the probability of latent human errors, psychological screening of health‐workers could provide valuable information to design staff reorganization.

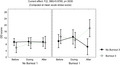

## 1 INTRODUCTION

First responders to epidemics are vulnerable to psychological distress, with influence from the potential to be both victim and service provider (Brooks et al., 2016). The most severe disorder elicited by exceptionally stressful life events is post‐traumatic stress disorder (PTSD) (APA, 2013), which has been related to depersonalization (altered perceptions of the self and the environment) (Spiegel, 1997). On the contrary, the ability to adapt successfully in the face of adversity (resilience) may be protective to most people (Bonanno & Mancini, 2012).

The psychological impact of the COVID‐19 pandemic on frontline health workers has been assessed around the world. However, the majority of studies available are cross‐sectional surveys that were performed remotely. In Italy, a web‐based cross‐sectional study on 1,379 health workers showed that younger age and female gender were related to adverse reactions (Rossi et al., 2020), with symptoms of post‐traumatic stress in 49.3% of respondents, severe depression in 24.7%, anxiety in 8.2%, insomnia in 19.8%, and high perceived stress in 21.9%. In Spain, an online survey was conducted on 1,422 health workers (1,228 women), displaying that emotional exhaustion and depersonalization contribute to psychological distress, while resilience and personal fulfillment can be protective factors (Luceño‐Moreno et al., 2020), with symptoms of post‐traumatic stress disorder in 56.6% of the respondents. In the United States of America, during a peak of inpatient admissions, a cross‐sectional web survey conducted on 657 physicians and nurses showed that especially nurses and advanced practice providers were experiencing psychological distress (Shechter et al., 2020), with 57% positive screen for acute stress, 48% for depressive symptoms, and 33% for anxiety symptoms. A systematic review and meta‐analysis to examine the pooled prevalence of depression, anxiety, and insomnia on 33,062 health workers, who participated in 13 cross‐sectional studies (mainly in China), showed female gender and nurses exhibiting higher rates of affective symptoms, compared to male gender and physicians, respectively (Pappa et al., 2020), with a prevalence of symptoms of anxiety and depression higher than 20%, and a prevalence of sleeping difficulties of 38.9%. A systematic review of 117 studies (65% conducted in Asian countries), including 119, 189 participants, also supports that younger age and female gender are risk factors for psychological distress during an infectious outbreak (Serrano‐Ripoll et al., 2020), with a pooled prevalence of 40% for acute stress (6,949 participants), 30% for anxiety (43,751 participants), 28% for burnout (1,168 participants), 24% for depression (61,463 participants), and 13% for post‐traumatic stress disorder (24,540 participants).

During epidemics, risk factors for adverse psychological reactions can be related to both context and individual factors (Brooks et al., 2018; Kisely et al., 2020). During the COVID‐19 pandemic, several context factors have been related to stress and burnout (Raudenská et al., 2020), including limited resources, threat of exposure to the virus, longer shifts, sleep disruption, and work‐life balance with increased workload. However, the main individual factors related to psychological distress include age, gender, and occupation (Luceño‐Moreno et al., 2020; Pappa et al., 2020).

We designed a follow‐up study of health workers at the frontline of COVID‐19 patient care, within the same working conditions, to assess the influence of their general characteristics and pre‐existing anxiety/depression/dissociative symptoms and resilience on the development of symptoms of post‐traumatic stress disorder (PTSD), while monitoring their quality of sleep, depersonalization/derealization symptoms (DD), acute stress, state anxiety, and burnout.

## 2 METHODS

### 2.1 Participants

The study protocol was approved by the institutional Research and Ethic Committees (R2020‐3602‐042), and written informed consent was obtained from all participants. Candidates were eligible if they were healthcare workers in a Hospital reconfigured to address the surge of patients with COVID‐19, at the Departments of Emergency Care, Intensive Care Unit, Internal Medicine, Respiratory Support, Imaging and Clinical Laboratory, including clinical, technical, and support workers.

After a standardized personal invitation to participate, among 261 candidates showing interest, 237 health workers accepted to participate; however, 204 (86.1%) completed the study protocol and gave answer to all the questionnaires at the 3 evaluations (Appendix S1). The 33 (13.9%) health workers who accepted to participate, but did not complete the study protocol, were 26–58 years old (mean age 34.3 years), 23 were women, and 10 were men, and they were working in clinical areas (*n* = 22), laboratory and imaging (*n* = 4), and support areas (*n* = 2). Among them, five workers were absent from the Hospital during the peak of inpatient admissions, due to sick‐leave (*n* = 3) or vacations (*n* = 2); three were transferred to another Hospital; one died (COVID‐19); 18 responded incomplete questionnaires; and five workers drop out of the study before the peak of inpatient admissions, while one drop out after the peak of admissions (Appendix S1).

Among the 204 participants (92 men/112 women; 19–58 years old), 128 (62.7%) were clinical staff, 120 clinicians (37 senior/83 junior) and 8 nurses (occupation 1); 56 (27.4%) were laboratory and imaging personnel (occupation 2); and 20 (9.8%) were support personnel (occupation 3). Of note, 27 (13.2%) were smokers, 94 (46%) reported alcohol consumption > 1/week, and 11 (5.4%) self‐reported physical or psychological disease. At the first evaluation, 2 (0.9%) participants already had been in quarantine due to COVID‐19 infection and, at the third evaluation, 36 (18.1%) participants had been infected.

### 2.2 Procedures

The study protocol included three evaluations: (1) The first evaluation was performed, while clinical spaces were reconfigured for COVID‐19 and before clinical teams were reorganized, (2) the second one, during the peak of inpatient admissions, and (3) the last one, just before clinical spaces were opened again for patients with diseases other than COVID‐19.

In the first evaluation, we performed a short assessment on demographics, contact with patients or biological samples of COVID‐19, and a medical history. Then, a psychological screening was performed using the following self‐administered questionnaires: Hospital Anxiety and Depression Scale (HADS) by Zigmond and Snaith (1983), Dissociative Experiences Scale (DES) by Bernstein and Putnam (1986) and Resilience scale by Connor et al. (2003).

The follow‐up of the participants was performed by administering the next inventories at the 3 time points: Pittsburgh Sleep Quality Index by Buysse et al. (1989), Depersonalization/derealization inventory (DD) by Cox and Swinson (2002), Stanford Acute Stress Questionnaire by Cardena et al. (2000), the short‐form of the State‐Trait Anxiety Inventory (STAIsv) by Marteau and Bekker (1992) and the short version of the Burnout Measure by Malach‐Pines (2005). At the end of the third evaluation, we also administered the Posttraumatic Stress Disorder Symptom Severity Scale‐Revised by Echeburúa et al. (2016). After each of the three evaluations, a psychiatrist reviewed all the questionnaires, using the accepted criteria for each instrument.

### 2.3 Analysis

Statistical analysis was performed using STATISTICA software (StatSoft Inc.). The significance level was set at 2‐tailed *α* = 0.05. We assessed data distribution using Kolmogorov–Smirnov test; since the scores of the inventories were not normally distributed, they are presented as medians with the first and third quartiles (Q1‐Q3). To assess linear correlations among the inventory scores, we used Pearson's correlation coefficient. To assess variations through time on the proportion of participants with bad quality of sleep and those with burnout, we used the Cochran *Q* test. To assess variations through time on the scores of the DD inventory, the acute stress questionnaire and the STAIsv, considering influence from age, gender, and occupation, we used repeated measures multivariate analysis of covariance. To assess the influence of age, gender, and occupation on the scores of the HADS, DES, and Resilience scale, as well as their influence on the PTSD score, considering the inventories administered at each of the 3 time points of evaluation, we performed multivariable regression analyses, using a generalized linear model (with log transformation) and Wald test.

### 2.4 Ethical approval

The study protocol was approved by the Local Institutional Research and Ethics Committees (R‐2020‐3601‐042). The study protocol was conducted in accordance with the Declaration of Helsinki and its amendments.

## 3 RESULTS

### 3.1 Psychological screening before follow‐up

Table 1 shows the inventory scores of the 204 participants who completed the study protocol. Noteworthy, the 33 health workers who did not complete the study protocol showed similar scores than those observed on the 204 participants, with median total score on the HADS of 11 (Q1–Q3 = 4–17.5), on the DES of 3.5 (1.9–7.8), and on the Resilience scale of 76 (68–85).

**TABLE 1:** Median and quartiles 1 & 3 of the inventory scores that were administered to 204 health workers, (1) before, (2) during, and (3) after the peak of inpatient admissions

Variables	Evaluation 1 Median (Q1‐Q3)	Evaluation 2 Median (Q1‐Q3)	Evaluation 3 Median (Q1‐Q3)
**Psychological screening**			
Hospital Anxiety and Depression Scale			
Anxiety score (all)	6.5 (3–10.5)	—	—
Women	7 (4–11)	—	—
Men	6 (2–10)	—	—
Depression score (all)	2 (1–5)	—	—
Women	3 (1–6)	—	—
Men	2 (0.5–4)	—	—
Total score (all)	8.5 (4–16)	—	—
Women	10 (5–17)	—	—
Men	7 (4–14)	—	—
Dissociative Experiences Scale (all)	6.2 (3.2–11.7)	—	—
Women	6.7 (3.2–11.7)	—	—
Men	5 (3.5–11.4)	—	—
Resilience scale (all)	78 (69.5–86.5)	—	—
Women	76 (68–84)	—	—
Men	80 (73–88)	—	—
**Follow‐up**			
Pittsburgh Sleep Quality Index (all)	8 (5–10)	8 (6–12)	8 (5–11)
Women	8.5 (6–11)	9 (6–12)	9 (5–12)
Men	6 (5–9)	8 (5–11)	7 (4–10)
Depersonalization/derealization inventory (all)	5 (2–12)	4.5 (1–14)	4 (1–12)
Women	6.5 (2–12)	6 (2–15)	6 (2–15)
Men	4 (1–10)	4 (0–12)	3 (0–10)
Stanford Acute Stress Questionnaire (all)	17.5 (4.5–39.5)	14 (2–40.5)	11 (0–36.5)
Women	20.5 (7.5–47)	16.5 (3–47.5)	16.5 (3–47.5)
Men	12 (1.5–29)	10 (1–29)	6 (0–24)
State‐Trait Anxiety Inventory s.v. (all)	5 (3–7.5)	6 (3.5–10)	5 (3–8)
Women	6 (3–9)	7.5 (4–12)	6 (3–8)
Men	4.5 (2−6)	6 (3–9)	5 (2–6.5)
Burnout Measure (all)	2.1 (1.5–2.8)	2.2 (1.6–3.3)	2.1 (1.5–3)
Women	2.2 (1.6–3)	2.5 (1.7–3.4)	2.2 (1.6–3.3)
Men	2 (1.4–2.5)	1.9 (1.4–2.8)	1.9 (1.3–2.7)
Posttraumatic Stress Disorder Severity Scale (all)	—	—	1 (0–10.5)
Women	—	—	2.5 (0–16)
Men	—	—	0 (0–7.5)

Table 2 shows the results of the regression analysis on the influence of gender, age, and occupation of the 204 participants on the score of the HADS, DES, and Resilience scale. There was no independent influence from gender on any of the three scales, while the age of the participants had influence on the scores of the three scales, with a negative relationship with HADS and DES and a positive relationship with Resilience; and occupation had influence on the DES, with higher scores on support workers than clinical workers. In addition, the interaction between gender and occupation had influence on the scores of the Resilience scale and DES among women, and just on the DES among men.

**TABLE 2:** Results of the regression analysis on the scores of the Hospital Anxiety and Depression Scale (HADS), the Dissociative Experiences Scale (DES), and the Resilience scale, including the age, gender, and occupation of the 204 participants

Variables	HADS *Estimate ± SE*	DES *Estimate ± SE*	Resilience Scale *Estimate ± SE*
Intercept	3.037 ± 0.26	3.46 ± 0.36	4.179 ± 0.056
Wald statistic (*p* value)	132.33 (<**.000001**)	89.61(<**.000001**)	5,469 (<**.000001**)
Age	−0.019 ± 0.007	−0.031 ± 0.011	0.0043 ± 0.001
Wald statistic (*p* value)	6.50 (**.010**)	7.94 (**.004)**	8.08 (**.004**)
Gender	0.055 ± 0.063	−0.09 ± 0.07	0.003 ± 0.016
Wald statistic (*p* value)	0.76 (.38)	1.84 (.17)	0.036 (.84)
Occupation (clinical versus support)	−0.088 ± 0.077	−0.418 ± 0.09	0.020 ± 0.019
Wald statistic (*p* value)	1.30 (.25)	17.84 (**.00002**)	1.085 (.29)
Occupation (technical versus support)	−0.038 ± 0.087	−0.026 ± 0.104	0.006 ± 0.021
Wald statistic (*p* value)	0.19 (.66)	0.063 (.80)	0.081 (.77)
Occupation*gender (women)	0.139 ± 0.074	0.237 ± 0.095	0.037 ± 0.01
Wald statistic (*p* value)	3.55 (.059)	6.18 (**.012**)	3.99 (**.045**)
Occupation*gender (men)	−0.152 ± 0.087	−0.106 ± 0.104	0.005 ± 0.02
Wald statistic (*p* value)	3.05 (.08)	1.04 (**<.000001**)	0.076 (.78)

Note

Coefficient estimates and standard error (*SE*) of the estimates are described with Wald statistic and the *p* values.

### 3.2 Follow‐up assessments

During the three evaluations, good correlations were observed among the scores of all the inventories, including those administered for psychological screening and those administered during follow‐up (Appendix S2).

In the three evaluations, the quality of sleep was bad (score > 5) in the majority of participants, with good correlation among the scores obtained during each evaluation (Pearson's *r* from 0.58 to 0.61, *p* < .0001; Appendix S2). Nonetheless, during the peak of hospital admissions, the proportion of participants with bad quality of sleep was the highest (Cochran's *Q* test, *Q*(2) = 7.17, *p *= .02): 149 (73%, 95% CI 69.9%–76.1%) in the first evaluation; 157 (76.9%, 95% CI 74%–79.8%) in the second evaluation; and 139 (68.1%, 95% CI 64.9%–71.3%) in the third evaluation.

Good correlation was also observed among the burnout measure scores (Pearson's *r* from 0.58–0.66, *p *< .0001; Appendix S2). However, over the three evaluations, just small variations were observed on the frequency of burnout (score ≥ 3.5) (Cochran's *Q* test, *p* > .05): 31 (15.1%, 95% CI 12.6%–17.6%) in the first evaluation, 40 (19.6%, 95% CI 16.9%–22.3%) in the second evaluation, and 38 (18.6%, 95% CI 15.9%–21.3%) in the third evaluation.

**FIGURE 1:**
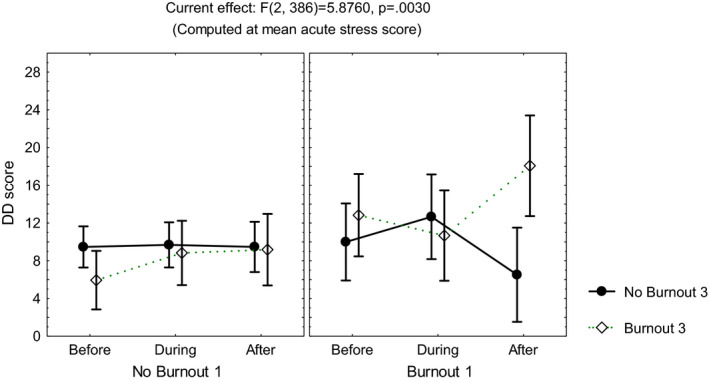
Mean and standard error of the mean of the score on the depersonalization/derealization (DD) inventory of 204 healthcare workers, according to burnout before and after the peak of inpatients admissions, computed at mean acute stress score

**FIGURE 2:**
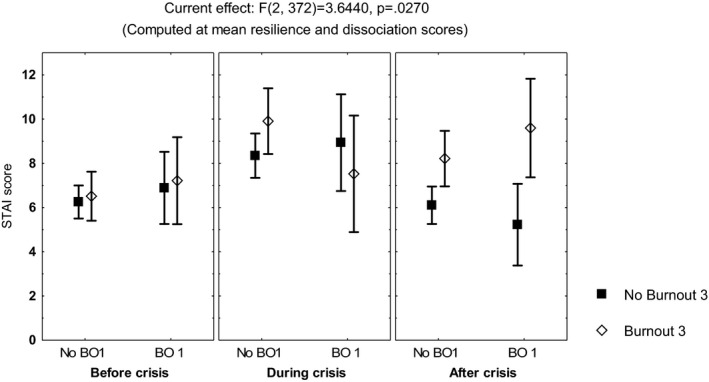
Mean and standard error of the mean of the score on the short‐form of the State‐Trait Anxiety Inventory (STAIsv) of 204 healthcare workers, according to burnout before and after the peak of inpatients admissions, computed at mean scores on the Resilience scale and the Dissociative Experiences Scale (DES)

**FIGURE 3:**
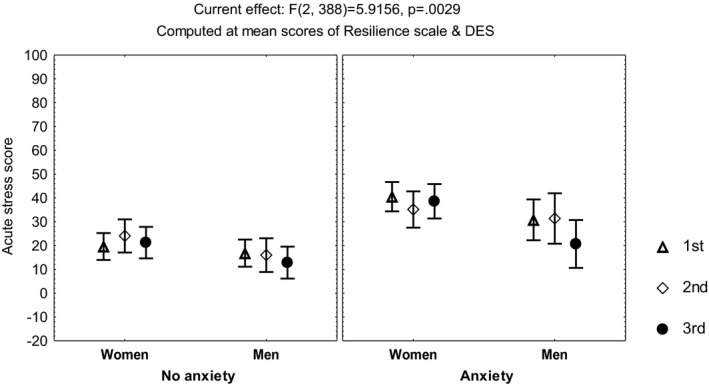
Mean and standard error of the mean of the score on the acute stress questionnaire of 204 healthcare workers, according to gender and anxiety, before and after the peak of inpatients admissions, computed at mean scores on the Resilience scale and the Dissociative Experiences Scale (DES)

Repeated measures multivariate analyses on the scores of the sleep quality index, the STAI, the DD inventory, the acute stress questionnaire, and the burnout measure, including age, gender, and occupation, showed no differences among the three time points of evaluation (MANCOVA, *p *> .05). However, influence from age was observed on the scores of the STAIsv, the DD inventory, the acute stress questionnaire, and the burnout measure (MANCOVA, *F*(1, 390) ≥4.3, *p *< .04), while influence from occupation was observed on the acute stress questionnaire (MANCOVA, *F*(3, 390) = 3.42, *p *< .01) and the DD inventory (MANCOVA, *F*(3, 390) = 5.7, *p *< .0008). Additionally, the highest scores on the DD inventory (Figure 1) and the STAIsv (Figure 2) were observed at the third evaluation, in the participants showing persistent burnout from the first to the third evaluations (MANCOVA, *F*(2, 386) = 5.87, *p *= .003 for the DD inventory, and *F*(2, 372) = 2.37, *p *= .02 for the STAIsv). At the three evaluations, the highest scores on the acute stress questionnaire were observed in women with anxiety symptoms (MANCOVA, *F*(2, 388) = 5.94, *p *= .002; Figure 3).

### 3.3 Post‐traumatic stress disorder

Thirty‐five participants (17.1%, 95% CI 14.5%–19.7%) fulfilled the criteria of the Posttraumatic Stress Disorder Symptom Severity Scale‐Revised (Echeburúa et al., 2016), validated by a psychiatrist according to DSM‐5 (APA, 2013).

Table 3 shows the results of the regression analysis to assess the influence of gender, age, occupation, and the scores on the HADS, DES, and Resilience scale on PTDS, while taking into account the score on each of the 3 sets of questionnaires administered during follow‐up (quality of sleep index, STAIsv, DD inventory, acute stress questionnaire, and burnout measure):

- At the first evaluation, the PTSD score was negatively related to the age of the participants and positively related to the total scores on the HADS, DES, STAIsv, DD inventory, and acute stress questionnaire (Wald test, intercept estimate −2.26 ± standard error 0.88, Wald Statistic = 16.61, *p *= .00004).
- At the second evaluation, the PTSD score was related to occupation, where laboratory and imaging personnel had the highest scores (median 5, Q1–Q3 = 0–13); it also was related to the scores on the previously administered HADS, DES, and Resilience scale and to the scores obtained at the second evaluation on the quality of sleep index, STAIsv, and acute stress questionnaire (Wald test, intercept estimate −4.87 ± 0.68, Wald Statistic = 6.5, *p *= .01).
- At the third evaluation, the PTSD score showed no relationship with gender, age or occupation but with the previously administered HADS and Resilience scale, as well as to the scores obtained at the third evaluation on the STAIsv and the acute stress questionnaire (Wald test, intercept estimate −4.36 ± 1.07, Wald Statistic = 51.34, *p* < .00001).

**TABLE 3:** Results of the regression analysis on the evidence of post‐traumatic stress disorder after the peak of inpatient admissions; including the general characteristics of the 204 participants, the scores on the psychological screening inventories, and the scores on the questionnaires administered (1) before, (2) during, and (3) after the peak of inpatient admissions

Variables	Evaluation 1 *Estimate ± SE*	Evaluation 2 *Estimate ± SE*	Evaluation 3 *Estimate ± SE*
**Psychological screening**			
Hospital Anxiety and Depression Scale	0.105 ± 0.030	0.084 ± 0.018	0.023 ± 0.010
Wald statistic (*p* value)	12.14 (**.0004**)	20.81 (**.000005**)	4.85 (**.02**)
Dissociative Experiences Scale	−0.095 ± 0.020	−0.040 ± 0.010	−0.000 ± 0.008
Wald statistic (*p* value)	21.25 (**.000004**)	14.92 (**.0001**)	0.002 (.95)
Resilience Scale	−0.007 ± 0.007	−0.015 ± 0.006	0.010 ± 0.004
Wald statistic (*p* value)	0.85 (.35)	4.72 (**.02**)	6.65 (**.009**)
**Follow‐up**			
Pittsburgh Sleep Quality Index	0.005 ± 0.042	0.076 ± 0.038	0.007 ± 0.033
Wald statistic (*p* value)	0.02 (.88)	3.92 (**.047**)	0.05 (.81)
Depersonalization/derealization scale	0.084 ± 0.026	0.009 ± 0.009	0.005 ± 0.005
Wald statistic (*p* value)	10.12 (**.001**)	1.10 (**.29**)	1.22 (.26)
Stanford Acute Stress questionnaire	0.018 ± 0.004	0.017 ± 0.005	0.012 ± 0.003
Wald statistic (*p* value)	16.56 (**.00004**)	12.03 (**.0005**)	15.38 (**.00008**)
State‐Trait Anxiety Inventory	−0.187 ± 0.054	−0.111 ± 0.025	0.128 ± 0.028
Wald statistic (*p* value)	10.12 (**.001**)	19.50 (**.00001**)	20.90 (**.000004**)
Burnout Measure	0.178 ± 0.126	0.064 ± 0.111	0.150 ± 0.082
Wald statistic (*p* value)	2.00 (.15)	0.33 (.56)	3.28 (.06)
**General characteristics**			
Gender	−0.062 ± 0.178	−0.197 ± 0.370	0.208 ± 0.165
Wald statistic (*p* value)	0.122 (.72)	0.28 (.59)	1.59 (.20)
Age	0.040 ± 0.019	−0.006 ± 0.012	−0.001 ± 0.009
Wald statistic (*p* value)	4.29 (**.038**)	0.25 (.61)	0.01 (.88)
Occupation			
Clinical & support categories	−0.062 ± 0.198	−0.050 ± 0.379	−0.051 ± 0.164
Wald statistic (*p* value)	0.10 (.75)	0.017(.89)	0.09 (.75)
Technical & support categories	−0.065 ± 0.194	0.788 ± 0.385	0.163 ± 0.123
Wald statistic (*p* value)	0.11 (.73)	4.17 (**.040**)	1.77 (.18)

Note

The coefficient estimates and standard error (*SE*) of the estimates are shown with the Wald statistic and the *p* values.

## 4 DISCUSSION

The results of this follow‐up study support that pre‐existing anxiety/depression and dissociation symptoms may contribute to the development of PTSD symptoms in frontline health workers, during a peak of inpatients admissions at COVID‐19 patient care, with protective influence from pre‐existing resilience, while persistent burnout may contribute to depersonalization symptoms and acute stress. In addition, before, during, and after the epidemic crisis, women with anxiety symptoms may be more prone to acute stress than men.

Burnout and stress among healthcare workers have been associated with negative impacts on the individual, patients and healthcare system (Walton et al., 2020; West et al., 2018). In this study, several factors may have contributed to a lower frequency of burnout and PTSD, compared to reports from a variety of countries (Dobson et al., 2020; Luceño‐Moreno et al., 2020; Matsuo et al., 2020). In this study, the scores on the resilience scale were high, with an inverse relationship with all the scales of psychological distress. The study was performed in a Hospital reorganized to address the surge of patients with COVID‐19, including balance between shifts and resting time, as well as task assignment according to professional profile, which could have reduced both stress and burnout (for a review see Walton et al., 2020). Another contributing factor could have been that participants knew that their responses to the questionnaires were evaluated at each time‐point during follow‐up, and psychiatric recommendations were provided when required. In addition, according to a large systematic review, during the COVID‐19 pandemic, PTSD, anxiety, and burnout are more likely to develop in nurses (Serrano‐Ripoll et al., 2020), and among the participants of this study, nurses were a minority.

In this study, the frequency of bad quality of sleep was the highest during the peak of hospital admissions, with influence on the development of PTSD symptoms. However, even before the peak in admissions, circa 70% of participants had bad quality of sleep. These findings are consistent with the high frequency of sleep disturbance found among workers with long working hours (Afonso et al., 2017), and particularly among clinical workers (Gómez‐García et al., 2016; Stewart & Arora, 2019). Although this factor might be modifiable, any operative strategy would require cultural, organizational, educational, and individual efforts.

The main limitation of this study was the recruitment from a single hospital, which may not be representative of other hospitals. However, this setting endorsed control for the working conditions and direct interaction with the participants. A second limitation of the study was the sample size, which allowed us to address just the more evident relationships among the study variables. In addition, the time frame of the study was not enough to allow assessment of long‐term mental health outcomes. Another limitation was the reliance on self‐report; since the study was designed to assess psychological reactions of busy workers, we had to rely on a selected set of self‐administered questionnaires, which were reviewed by the same psychiatrist; besides, since the Hospital was reorganized for the surge of patients with COVID‐19, no adequate controls were available to assess changes over time; however, the stability of the follow‐up measurements supports that the findings were not just time related.

## 5 CONCLUSIONS

In health workers at the frontline of COVID‐19 patient care, within the same environment and working circumstances, the assessment of the influence of individual predisposing factors to adverse psychological reactions to peak inpatient admissions showed that:

- Pre‐existent anxiety/depression, dissociative symptoms, as well as coexisting acute anxiety and acute stress contribute to PTSD symptoms.
- Persistent burnout may contribute to state anxiety, acute stress, and depersonalization symptoms.
- Compared to men, women reporting pre‐existent anxiety may be more prone to acute stress.
- The quality of sleep of healthcare workers may be bad, even before the epidemic crisis, and possibly contribute to stress.
- Younger age could be related to pre‐existent common psychological symptoms and less resilience.
- Pre‐existent resilience may have favorable influence both during and after an epidemic crisis.

During an infectious outbreak, in order to protect the mental health of vulnerable personnel and decrease the probability of latent human errors, psychological screening could provide valuable information to prevent or mitigate against adverse psychological reactions by frontline healthcare workers caring for patients.

## CONFLICT OF INTEREST

All authors report no disclosures relevant to the manuscript.

## AUTHOR CONTRIBUTION

JAMP and DCB executed the research project; were data curators; and reviewed/critiqued the data analysis and the manuscript. FJAG, LASH, TCM, EEP, JCAG, JIGD, OBSS, and LCVA executed the research; organized the data; and reviewed/critiqued manuscript preparation. KJR conceived, organized, and executed research project; designed and executed data analysis; and first drafted and reviewed/critiqued the manuscript.

## ETHICAL APPROVAL

The study protocol was approved by the Local Institutional Research and Ethics Committees. The study protocol was conducted in accordance with the Declaration of Helsinki and its amendments.

### Peer Review

The peer review history for this article is available at https://publons.com/publon/10.1002/brb3.2007.

## Supporting information

Appendix S1

Appendix S2

## Data Availability

Data available at: https://dataverse.harvard.edu/dataset.xhtml?persistentId=doi:10.7910/DVN/ZO5OXH
