# Supplementary material for: Burnout, depersonalization, and anxiety contribute to post‐traumatic stress in frontline health workers at COVID‐19 patient care, a follow‐up study
Source: Brain Behav. 2020 Dec 15;11(3):e02007. doi: 10.1002/brb3.2007 (PMC7883101; doi:10.1002/brb3.2007)
Supplement: Supplementary file 1 — Appendix S1 [file BRB3-11-e02007-s002.docx]

**Appendix 1.**

3-relocated

**AFTER CRISIS**

205 participants

1- incomplete questionnaires

**204 participants completed the protocol**

261 candidates to participate

**DURING CRISIS**

1- drop out

5- drop out

2- vacations

227 participants

3- sick-leave

(2 COVID-19)

1- dead

(COVID-19)

17- incomplete questionnaires

237 participants

**BEFORE CRISIS**
