# Supplementary material for: Burnout, depersonalization, and anxiety contribute to post‐traumatic stress in frontline health workers at COVID‐19 patient care, a follow‐up study
Source: Brain Behav. 2020 Dec 15;11(3):e02007. doi: 10.1002/brb3.2007 (PMC7883101; doi:10.1002/brb3.2007)
Supplement: Supplementary file 2 — Appendix S2 [file BRB3-11-e02007-s001.docx]

Appendix 2.

| **A** | Psychological screening | | | | | **Evaluation 1** | | | | | **Evaluation 2** | | | | | **Evaluation 3** | | | | | |
| --- | --- | --- | --- | --- | --- | --- | --- | --- | --- | --- | --- | --- | --- | --- | --- | --- | --- | --- | --- | --- | --- |
| **Inventory** | Resilience | HADS  A* | HADS  D* | HADS  T* | DES | Sleep quality | State anxiety | DD* | Acute stress | Burnout | Sleep quality | State anxiety | DD* | Acute stress | Burnout | Sleep quality | State anxiety | DD* | Acute stress | Burnout | PTSD* |
| Resilience | 1 | -0.45 | -0.57 | -0.54 | -0.38 | -0.3 | -0.49 | -0.48 | -0.429 | -0.557 | -0.3 | -0.351 | -0.294 | -0.26 | -0.39 | -0.27 | -0.44 | -0.36 | -0.28 | -0.3911 | -0.362 |
|  | *p= ---* | *p=.00* | *p=.00* | *p=.00* | *p=.000* | *p=.000* | *p=.000* | *p=.00* | *p=.000* | *p=.000* | *p=.000* | *p=.000* | *p=.000* | *p=.000* | *p=.000* | *p=.00* | *p=.000* | *p=.00* | *p=.000* | *p=.000* | *p=.00* |
| HADS A | -0.45 | 1 | 0.72 | 0.95 | 0.484 | 0.557 | 0.791 | 0.579 | 0.6587 | 0.6211 | 0.4855 | 0.586 | 0.4058 | 0.481 | 0.54 | 0.352 | 0.558 | 0.426 | 0.44 | 0.5312 | 0.424 |
|  | *p=.000* | *p= ---* | *p=0.0* | *p=0.0* | *p=.000* | *p=.000* | *p=0.00* | *p=.00* | *p=.000* | *p=.000* | *p=.000* | *p=.000* | *p=.000* | *p=.000* | *p=.000* | *p=.00* | *p=.000* | *p=.00* | *p=.000* | *p=.000* | *p=.00* |
| HADS D | -0.57 | 0.72 | 1 | 0.9 | 0.481 | 0.445 | 0.688 | 0.574 | 0.6677 | 0.6004 | 0.4271 | 0.4197 | 0.4263 | 0.453 | 0.513 | 0.292 | 0.478 | 0.439 | 0.43 | 0.4198 | 0.439 |
|  | *p=.000* | *p=0.00* | *p= ---* | *p=0.0* | *p=.000* | *p=.000* | *p=.000* | *p=.00* | *p=.000* | *p=.000* | *p=.000* | *p=.000* | *p=.000* | *p=.000* | *p=.000* | *p=.00* | *p=.000* | *p=.00* | *p=.000* | *p=.000* | *p=.00* |
| HADS T | -0.54 | 0.95 | 0.9 | 1 | 0.519 | 0.549 | 0.805 | 0.621 | 0.713 | 0.6592 | 0.4962 | 0.5557 | 0.446 | 0.505 | 0.569 | 0.352 | 0.565 | 0.464 | 0.469 | 0.5215 | .4634 |
|  | *p=.000* | *p=0.0* | *p=0.0* | *p= ---* | *p=.000* | *p=.000* | *p=0.00* | *p=.00* | *p=0.00* | *p=.000* | *p=.000* | *p=.000* | *p=.000* | *p=.000* | *p=.000* | *p=.00* | *p=.000* | *p=.00* | *p=.000* | *p=.000* | *p=.00* |
| DES | -0.38 | 0.48 | 0.48 | 0.52 | 1 | 0.358 | 0.377 | 0.796 | 0.5465 | 0.4927 | 0.341 | 0.3024 | 0.6101 | 0.459 | 0.425 | 0.274 | 0.275 | 0.564 | 0.434 | 0.3329 | 0.441 |
|  | *p=.000* | *p=.00* | *p=.00* | *p=.00* | *p= ---* | *p=.000* | *p=.000* | *p=0.0* | *p=.000* | *p=.000* | *p=.000* | *p=.000* | *p=.000* | *p=.000* | *p=.000* | *p=.00* | *p=.000* | *p=.00* | *p=.000* | *p=.000* | *p=.00* |

Correlation among the inventory scores administered to 204 healthy workers, before (evaluation 1), during (evaluation 2) and after (evaluation 3) the peak of inpatient admissions of patients with COVID-19; including: (A) the psychological screening performed by a Resilience scale, the Hospital Anxiety and Depression Scale (*HADS, where A= anxiety, D= depression, T= total*), and the Dissociative Experiences Scale (DES) and the follow-up using the Sleep quality Index, the short form of the State-Trait Anxiety Inventory, a Depersonalization/Derealization inventory (DD), the Stanford Acute Stress Questionnaire, the short form of the Burnout measure and the Posttraumatic Stress Disorder Severity Scale (PTSD); as well as (B) the correlation among the follow-up inventories.

| **B** | **Evaluation 1** | | | | | **Evaluation 2** | | | | | **Evaluation 3** | | | | |
| --- | --- | --- | --- | --- | --- | --- | --- | --- | --- | --- | --- | --- | --- | --- | --- |
| **Inventory** | Sleep quality | State anxiety | DD | Acute stress | Burnout | Sleep quality | State anxiety | DD | Acute stress | Burnout | Sleep quality | State anxiety | DD | Acute stress | Burnout |
| Sleep quality | 1 | 0.55 | 0.485 | 0.58 | 0.588 | 1 | 0.6 | 0.53 | 0.57 | 0.621 | 1 | 0.6 | 0.51 | 0.55 | 0.612 |
|  | *p= ---* | *p=.000* | *p=.000* | *p=.000* | *p=.000* | *p= ---* | *p=.000* | *p=.000* | *p=.000* | *p=.000* | *p= ---* | *p=.000* | *p=.000* | *p=.000* | *p=.000* |
| State anxiety | 0.55 | 1 | 0.504 | 0.574 | 0.608 | 0.6 | 1 | 0.51 | 0.54 | 0.644 | 0.6 | 1 | 0.55 | 0.55 | 0.693 |
|  | *p=.000* | *p= ---* | *p=.000* | *p=.000* | *p=.000* | *p=.000* | *p= ---* | *p=.000* | *p=.000* | *p=.000* | *p=.000* | *p= ---* | *p=.000* | *p=.000* | *p=.000* |
| DD | 0.485 | 0.504 | 1 | 0.698 | 0.598 | 0.53 | 0.51 | 1 | 0.72 | 0.604 | 0.51 | 0.55 | 1 | 0.69 | 0.618 |
|  | *p=.000* | *p=.000* | *p= ---* | *p=0.00* | *p=.000* | *p=.000* | *p=.000* | *p= ---* | *p=0.00* | *p=.000* | *p=.000* | *p=.000* | *p= ---* | *p=.000* | *p=.000* |
| Acute stress | 0.58 | 0.574 | 0.698 | 1 | 0.676 | 0.57 | 0.54 | 0.72 | 1 | 0.644 | 0.55 | 0.55 | 0.69 | 1 | 0.582 |
|  | *p=.000* | *p=.000* | *p=0.00* | *p= ---* | *p=.000* | *p=.000* | *p=.000* | *p=0.00* | *p= ---* | *p=.000* | *p=.000* | *p=.000* | *p=.000* | *p= ---* | *p=.000* |
| Burnout | 0.588 | 0.608 | 0.598 | 0.676 | 1 | 0.62 | 0.64 | 0.6 | 0.64 | 1 | 0.61 | 0.69 | 0.62 | 0.58 | 1 |
|  | *p=.000* | *p=.000* | *p=.000* | *p=.000* | *p= ---* | *p=.000* | *p=.000* | *p=.000* | *p=.000* | *p= ---* | *p=.000* | *p=.000* | *p=.000* | *p=.000* | *p= ---* |
